# A Million for the Hospitals

**Published:** 1919-09-20

**Authors:** 


### The Hospital

The Workers' Newspaper of Administrative Medicine and Institutional Life, Administration, National Insurance and Health.

No. 1737, Vol. LXVI  SATURDAY, SEPTEMBER 20, 1919.  PRICE TWOPENCE

### A MILLION FOR THE HOSPITALS.

The winding-up of the accounts of the British Red Cross Society and the Order of St. John is a vast business, which is complicated by the heavy commitments still to be met out of the funds in hand, but the first great step has been taken by the Joint War Committee. This is its decision to allot, in the form of immediate grants, the sum of £956,900 to the hospitals and nursing associations of England and Wales. The Joint Committee has been led to this decision by the knowledge that those who have subscribed the vast sums entrusted to it will desire part of the surplus to be distributed for the immediate alleviation of sickness and suffering. That desire is certainly present, and when we study the method of distribution we understand afresh the immense work performed by the voluntary hospitals during the war. The Army Council, moreover, has sent a special letter of thanks to the Hon. Sir Arthur Stanley, Chairman of the Joint War Committee, in which "the inestimable service" of the Red Cross and the Order of St. John is recorded. A special tribute is paid to the "organisations and hospitals" formed in connection with the two societies, and the work done is said to have "contributed in no small measure" to the victory of the Allies. The praise of the Army Council, which corresponds to the thanks of Parliament lately given to the admirals, generals, fleets, and armies, is then to be accompanied by the award given by the Joint Committee, and that award is the practical recognition of the voluntary hospitals' work.

Under the terms of the Red Cross and St. John Act, 1918, the surplus of the funds subscribed to the joint societies for the benefit of sick and wounded soldiers can be used for the relief of sickness generally. It is proper, therefore, that the surplus should be bestowed on the institutions which came to the rescue by receiving soldiers in addition to their civilian patients. The effect will be to limit the first distribution to institutions which treat ex-Service men, and to exclude hospitals solely for women and children. How far the latter, in their treatment of dependants of soldiers and sailors, may be eligible for later distributions is not apparent. But the first distribution is confined to the hospitals which treat the fighting men themselves. The institutions have been chosen and the grants determined in the following way. At the request of the Joint Finance Committee the Central Demobilisation Board invited each county director, in consultation with the county demobilisation committee, to submit applications for grants for hospitals and institutions in the county before the end of May. During July and August Captain Colchester and Sir Frederick Fry examined in person individual applicants. The two assessors, so to call them, presented their report on September 3, and it was adopted by the Demobilisation Board, the Joint War Committee, and the Joint Finance Committee. The money was then voted.

The beneficiaries are divided into hospitals which treat ex-Service men only; voluntary hospitals, whose patients include ex-Service men; institutions which give ex-Service men priority, such as farm colonies for consumptives; and county district nursing associations. It should be noted that the grants made to voluntary hospitals and farm colonies are only for the encouragement of building schemes, extensions, and improvements. No money has been granted to endow or maintain beds, or to pay debts, while the grants to county nursing associations have been based on the population of the county served by the applying association.

In the case of the London hospitals a lump sum of £125,000 has been voted, the distribution of which King Edward's Hospital Fund Committee has been asked to undertake. In the case of the Provinces, three trustees have been appointed for each county, to whose account the money will be paid. These trustees have to be satisfied that the scheme for which the money is intended is mature. This means that either the balance needed to complete the scheme has been raised, or that a sum equal to the grant proposed by the Joint Committee has been subscribed. Each granter must sign a definite undertaking to apply the grant only to the precise purpose for which it is given. A further important condition is that the committees of all hospitals receiving a grant over £1,000 must prepare a full yearly audited balance sheet and statistics of patients and the cost of their treatment on the system adopted by all the London hospitals. Thus the uniform system of hospital accounts and the statistical returns of work done are now recognised officially to be a *sine qua non* of efficient administration. The grants are also conditioned by a clause which provides the county V.A.D. organisation shall be properly represented, if it so desires, on the committee of management of each institution in receipt of a grant. The trustees must pay all grants before August 31, 1920, any not paid must be returned to the Joint Finance Committee, and proper accounts must, of course, be kept by the trustees. It is to be noted that the grants are limited to England and Wales, because Scotland has its own funds. Ireland is allotted a separate sum for Irish hospitals. A list of grants is published under the names of the different counties. Jersey, to take a simple example, receives £500 for its County Nursing Association; Worcestershire, £15,000 for its farm colony for consumptives, Devon, £10,000 for the Royal Devon and Exeter Hospital, £20,000 for its farm colony, £3,000 for its nursing associations, and so on. As we glance down the list of counties, Worcester, for example, it is a surprise to see only one organisation, and that not the Worcester Infirmary, mentioned. Is the explanation that no application for the hospital was made, and if not, why not?

The County Directors of six English counties, the West Riding of Yorkshire, and one Welsh county appear not to have applied in accordance with the conditions under which grants have been made. The bestowal of these grants from Red Cross funds to different units of the voluntary hospital system unites the vast public, which responds to the appeal of charity under the impulse only of war, to the hospitals which depend on the ordinary impulse of generosity.

The supporters of war charities and peace charities are different. By this grant from Red Cross funds the two streams are directed into the voluntary hospital channel. Patriotism itself leads to the hospitals at last. This is now proved, and since the war lasted long enough to make patriotic generosity a habit, we urge once again that the organisation on which patriotic generosity depended should not be allowed to lapse, but that it should be continued if only because ex-Service men will long be numbered among hospital patients. The great public has now become actively interested in charity. It should be allowed to remain so, in order that the voluntary hospitals may be permanently supported on the wider base which was created in the war.

